# Alterations in BCR heavy chain CDR3 repertoire characteristics in pediatric mycoplasma pneumoniae infection

**DOI:** 10.3389/fcimb.2025.1573511

**Published:** 2025-06-12

**Authors:** Yanfei Chen, Yi Yuan, Xingzhu Liu, Bin Li, Lijuan Meng, Ying Xiao, Zhongjian Su, Linfei Han, Hong Li, Lili Deng, Jun Li, Caixia Ye, Xing Zhang

**Affiliations:** ^1^ Department of Cardiology, Kunming Children’s Hospital, Kunming, Yunnan, China; ^2^ Department of Immunology, Center of Immunomolecular Engineering, Innovation and Practice Base for Graduate Students Education, Zunyi Medical University, Zunyi, China; ^3^ Department of Pediatrics 1, Yunyang Maternal and Child Health Hospital, Chongqing, China

**Keywords:** Mycoplasma pneumoniae, B cell receptor, CDR3 repertoire, flow cytometry, clonal expansion

## Abstract

**Introduction:**

Mycoplasma pneumoniae (MP) infection is a leading cause of pediatric pneumonia, triggering a complex immune response in which B cells play a critical role. This study aimed to analyze B cell receptor (BCR) heavy chain CDR3 repertoires in MP patients.

**Methods:**

Clinical data from 202 children diagnosed with MP were retrospectively analyzed. Flow cytometry was used to assess B cell counts in 99 MP patients and 25 healthy controls (HC). Multiplex PCR was used to construct BCR heavy chain CDR3 repertoires from peripheral blood samples of 8 MP patients and 9 HC.

**Results:**

Serological analysis revealed elevated levels of inflammatory markers, including C-reactive protein, interleukin-6, and ferritin, indicating an active immune response. Flow cytometry showed significantly increased B cell counts in MP patients compared to HC. Immunoglobulin levels were elevated in several patients, indicating immune fluctuations during infection. BCR repertoire analysis revealed increased diversity and altered clonotype distribution in MP patients, with preferential usage of IGHV1-18, IGHV7-4-1, and IGHJ6. MP patients exhibited a bimodal distribution of CDR3 lengths, with significantly longer CDR3 regions. Sixty-eight MP-exclusive clonotypes were identified, with evidence of clonal expansion.

**Conclusion:**

These findings suggest that alterations in the BCR heavy chain CDR3 repertoire play a crucial role in the immune response to MP infection and may offer insight into disease progression and therapeutic targets.

## Introduction

Mycoplasma pneumoniae (MP) is a leading cause of community-acquired pneumonia in children, often resulting in significant morbidity and prolonged hospitalization (Kutty et al., 2019; Li et al., 2021). While MP infections are generally self-limiting, severe and refractory cases have been increasingly reported (Choi et al., 2022; Li et al., 2023), raising concerns about disease pathogenesis and immune response dynamics. Pediatric MP infections can manifest in a spectrum of clinical presentations, ranging from mild upper respiratory symptoms to severe pneumonia with extrapulmonary complications (Atkinson et al., 2008; Waites et al., 2017). The immune response to MP is a critical determinant of disease severity, with excessive inflammation and immune dysregulation contributing to more severe manifestations (Waites and Talkington, 2004; Zhu et al., 2023).

Humoral immunity, particularly the B cell response, plays a central role in host defense against infection, including those caused by bacteria (Fan et al., 2021), viruses (Miwa et al., 2023), and fungi (Casadevall et al., 2002). B cells mediate immunity through antibody production, antigen presentation, and cytokine secretion. The B cell receptor (BCR) repertoire, particularly the diversity and clonality of immunoglobulin heavy chain complementarity-determining region 3 (CDR3), provides insights into the adaptive immune response to infection (He et al., 2022; Kotagiri et al., 2022; Mai et al., 2023). The BCR repertoire is shaped by V(D)J recombination, somatic hypermutation, and clonal selection, generating a highly diverse set of antigen-specific receptors (Papavasiliou et al., 1997; Ollila and Vihinen, 2005; Spisak et al., 2024). This diversity enables the immune system to recognize and respond to a wide range of pathogens. The alterations in BCR repertoire characteristics, such as skewed V and J gene usage, abnormal CDR3 length distribution, and the presence of disease-specific clonotypes, may be associated with immune dysfunction and the host’s response to specific pathogens.

Despite the known involvement of B cells in MP infections (Zhang et al., 2022; Li et al., 2024), comprehensive analyses of the BCR heavy chain CDR3 repertoire in pediatric MP patients remain limited. Understanding the characteristics of the BCR repertoire in these patients could provide valuable insights into disease mechanisms, immune evasion strategies of MP, and potential biomarkers for disease severity. In this study, we analyzed the BCR heavy chain CDR3 repertoire in pediatric MP patients, focusing on serological characteristics, B cell activation, V and J gene usage, CDR3 length distribution, amino acid composition, and disease-specific clonotypes. Our findings offer new perspectives on the adaptive immune response to MP and contribute to a deeper understanding of immune dynamics in pediatric respiratory infections.

## Materials and methods

### Participant demographics and sample collection

This study aimed to examine the serological characteristics, B cell response, and BCR heavy chain CDR3 repertoire in pediatric patients diagnosed with MP infection. A total of 202 MP patients, aged 4–6 years, were enrolled from the pediatric department at Kunming Children’s Hospital in Yunnan Province, China. All participants were diagnosed with MP infection based on Mycoplasma pneumoniae nucleic acid testing and clinical symptoms.

Blood samples (2 mL) were collected in EDTA tubes from 8 participant during their hospital admission. Additionally, 9 healthy children, matched for age and sex, served as controls, with blood samples drawn at Kunming Children’s Hospital. Detailed clinical data, including age, sex, clinical manifestations, and laboratory parameters were retrospectively collected from the participants’ medical records. The study was approved by the Institutional Review Board (IRB) of Kunming Children’s Hospital (Approval No: 2023-03-246-K01), and written consent was obtained from the parents or guardians of all pediatric patients. This research adhered to the ethical principles set forth in the Declaration of Helsinki.

### RNA extraction, cDNA synthesis, and quality control

Total RNA were extracted using the Tiangen RNA extraction kit (Tiangen Biotech, China), following the manufacturer’s instructions. RNA was then reverse transcribed into cDNA libraries using a standard reverse transcription kit. The final cDNA were collected and stored at -80°C until further analysis. The concentration and purity of the extracted nucleic acids were assessed using a NanoDrop 2000 Spectrophotometer (Thermo Fisher Scientific, USA), and the RNA integrity was evaluated by electrophoresis on a 1% agarose gel. Only samples with an RNA integrity number (RIN) greater than 7.0 were used for library construction and sequencing.

### Library preparation and sequencing

Library preparation was performed using a multiplex PCR-based method. Briefly, cDNA samples were subjected to multiplex PCR amplification using primers designed to target the IGH CDR3 regions. The PCR products were then purified and assessed for quality before being used for library construction. After preparing the libraries, they were sent to BGI Genomics (Shenzhen, China) for next-generation sequencing. High-throughput sequencing was performed using an Illumina NovaSeq 6000 platform to generate paired-end 150-bp reads.

### Pre-processing of raw sequencing data

We utilized the cDNA library sequencing data in FASTQ format for the multiplex PCR-based BCR repertoire analysis. Initial quality assessment was performed using FastQC to evaluate the overall sequencing quality, checking for issues such as low-quality reads (phred score <20) and adapter contamination. Reads that failed the quality assessment were trimmed using Trimmomatic v.0.39 to remove unwanted sequences. After ensuring the data quality, we used MiXCR software to identify BCR clonotypes, converting raw sequencing data into quantitative clonotype information. The MiXCR analysis pipeline included aligning the sequencing reads to the V, D, J, and C genes of B cell receptors from the IMGT (International ImMunoGeneTics) database. The aligned reads were then assembled to extract the CDR3 gene regions, essential for BCR repertoire analysis. Clonotypes were defined based on identical CDR3 nucleotide sequences in combination with V and J gene usage, following the software’s default clonotype grouping parameters. To ensure quality and biological relevance, CDR3 amino acid sequences containing stop codons (“*”) or gaps (“-”) were excluded from downstream analysis. Each remaining clonotype thus represented a unique and productive BCR rearrangement. For further analysis, we utilized Immunarch to convert MiXCR’s output into a text file formatted for downstream analysis of BCR repertoire dynamics and diversity.

### Analysis of CDR3 sequences and diversity estimation

Comprehensive analyses of BCR CDR3 sequences were performed using the Immunarch package with default parameters. Key assessments included basic statistics, CDR3 length distribution, V(D)J gene segment usage, amino acid composition, clonotype richness, and evenness. BCR clonal diversity was estimated using D50 index. To identify conserved sequence motifs among the dominant MP-specific clonotypes, CDR3 amino acid sequences were submitted to the MEME Suite web server (https://meme-suite.org/meme/tools/meme). Motif discovery was conducted using default parameters, specifying the identification of up to three motifs.

### Data statistics and visualization

All statistical analyses were performed using R. The Wilcoxon rank-sum test was applied for non-parametric comparisons to assess statistical significance between groups, as the data either did not follow a normal distribution or had a small sample size. P-values < 0.05 were considered statistically significant. For data visualization, various plots, including boxplots and bar charts, were generated using the ggplot2 package in R. Significant p-values were manually annotated on the plots using the ggpubr package.

## Results

### Serological characteristics and activated B Cell response in pediatric MP patients

A total of 202 patients diagnosed with MP infection were included in the study. The clinical characteristics of MP patients are summarized in **Table 1**. Among them, 197 patients had available data on white blood cell count, lymphocyte count, monocyte count, lymphocyte percentage, and monocyte percentage. The mean WBC count was 8.7 ± 4.32 × 10^9^/L. The lymphocyte count averaged 3.15 ± 2.87 × 10^9^/L, while the monocyte count was 0.65 ± 0.39 × 10^9^/L. The lymphocyte percentage was 35.41 ± 14.3%, and the monocyte percentage was 7.88 ± 3.73%, both within their respective reference ranges. Inflammatory markers were also assessed. C-reactive protein levels were available for 171 patients, with a mean value of 10.86 ± 15.87 mg/L, slightly exceeding the upper reference limit. Serum interleukin-6 levels were elevated, with a mean of 11.49 ± 11.58 pg/ml in 129 patients. Ferritin levels, measured in 171 patients, averaged 148.43 ± 94.18 µg/L, exceeding the reference range. Complement levels were measured in a subset of patients, with C3 at 1.19 ± 0.26 g/L and C4 at 0.29 ± 0.11 g/L, both within reference ranges.

**Table 1:** Clinical characteristics of patients diagnosed with MP infection.

Test Item	Measured Value	Reference Range	Sample Size
White Blood Cell Count (10^9^/L)	8.7 ± 4.32	4.3-11.3	197
Lymphocyte Count (10^9^/L)	3.15 ± 2.87	1.5-4.6	197
Monocyte Count (10^9^/L)	0.65 ± 0.39	0.13-0.76	197
Lymphocyte Percentage (%)	35.41 ± 14.3	23-59	197
Monocyte Percentage (%)	7.88 ± 3.73	2-11	197
C-Reactive Protein (mg/L)	10.86 ± 15.87	0-10	171
Complement C4 (g/L)	0.29 ± 0.11	0.125-0.425	172
Complement C3 (g/L)	1.19 ± 0.26	0.8-1.5	157
Interleukin-6 (pg/ml)	11.49 ± 11.58	0-7	129
Ferritin (ug/L)	148.43 ± 94.18	7-142	171

**Figure 1:**
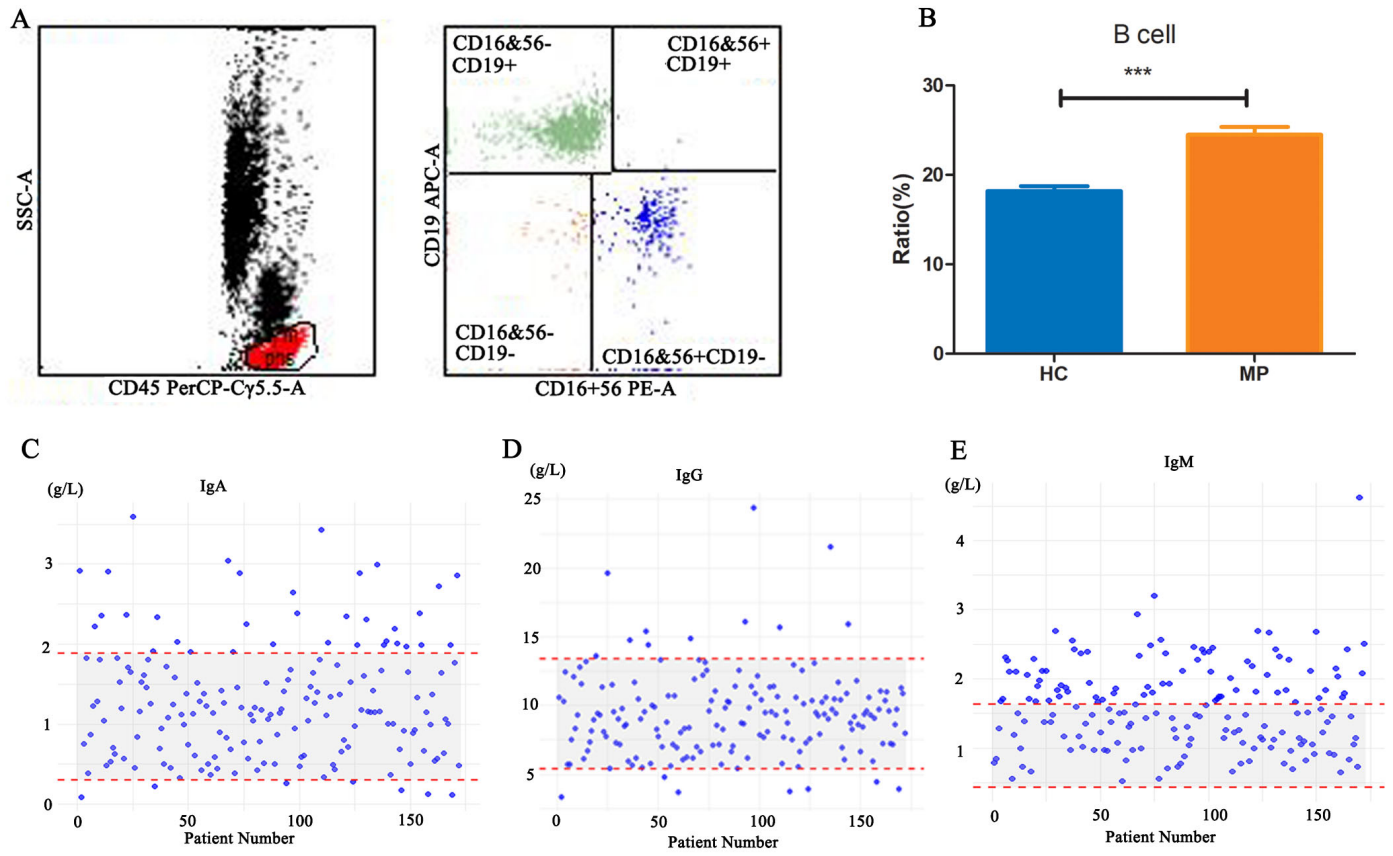
B cell counts and immunoglobulin levels in MP patients. **(A)** Representative flow cytometry gating strategy for identifying B cells in peripheral blood. **(B)** Comparison of peripheral blood B cell counts between MP patients (n=99) and healthy controls (HC, n=25). **(C-E)** Retrospective analysis of serum immunoglobulin levels (IgA, IgG, and IgM) in 173 MP patients. MP: Mycoplasma pneumoniae. HC: Healthy control. Data are presented as mean ± SEM. Statistical significance was determined using unpaired two-tailed Student’s t-test. ***p < 0.001.

The serological indicators of MP patients suggest an active state of immune response and inflammation. Flow cytometry analysis of peripheral blood B cell counts in 99 MP patients and 25 healthy children revealed that B cell numbers were significantly higher in MP patients compared to the HC group (**Figures 1A, B**), indicating that B cells may play an important role in the immune response during MP infection. A further retrospective analysis of immunoglobulin levels in 173 MP patients (**Figures 1C–E**) showed that the immunoglobulin levels were elevated in some patients, with 81, 34 and 11 patients having IgM, IgA, or IgG levels exceeding the reference range respectively. This suggests that MP infection may lead to abnormal fluctuations in immunoglobulin levels, providing further evidence for the involvement of B cells in the immune response to MP infection and potentially contributing to the progression of the disease.

### BCR heavy chain CDR3 repertoire analysis in pediatric MP patients

**Figure 2:**
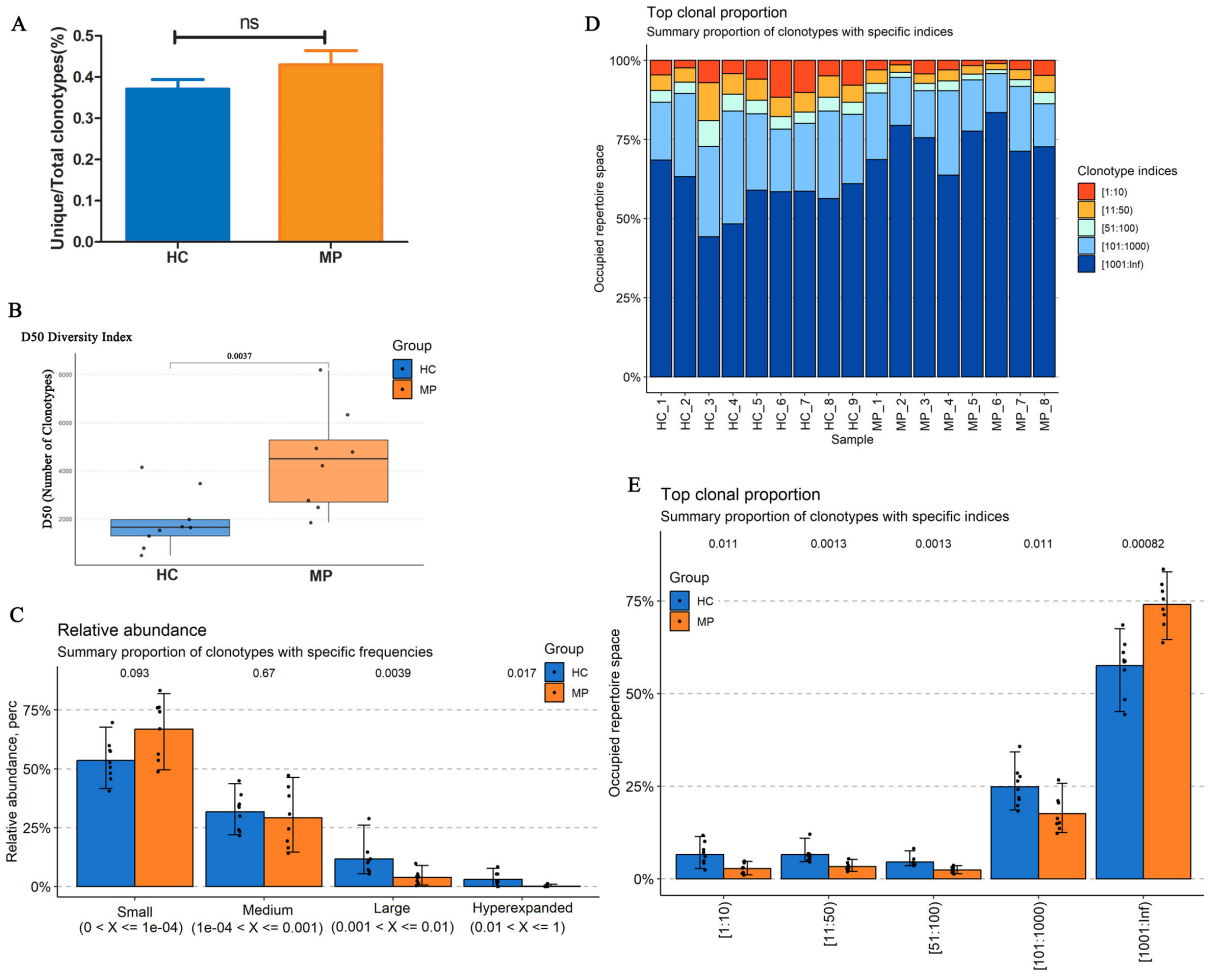
BCR heavy chain CDR3 repertoire diversity and clonotype distribution. **(A)** The proportion of unique BCR clones relative to the total clone count in MP and HC groups. **(B)** Diversity analysis revealed significantly increased BCR repertoire diversity in MP patients compared to HC. **(C)** Relative abundance distribution of BCR clonotypes. **(D)** Top clone analysis demonstrated a high degree of intra-group similarity. **(E)** Clonotype occupancy analysis. MP: Mycoplasma pneumoniae. HC: Healthy control. ns indicates no statistical difference. Data are presented as mean ± SEM. Statistical significance was determined using unpaired two-tailed Student’s t-test. ns indicates no statistical difference. P < 0.05 indicates statistical difference.

To further investigate BCR repertoire characteristics in pediatric MP patients, we analyzed the BCR heavy chain CDR3 repertoires from peripheral blood samples of eight MP patients and nine HC. After filtering the raw sequencing data, we randomly selected 100,000 clones from each sample for analysis. The proportion of unique clones relative to the total clone count was approximately 40% in both groups, with a higher percentage observed in the MP group compared to the HC group, though the difference was not statistically significant (**Figure 2A**).

Diversity analysis revealed a significantly higher diversity in the MP group than in the HC group (**Figure 2B**). Examination of the relative clone abundance indicated that both groups were predominantly composed of clones with Small and Medium frequencies (**Figure 2C**). Furthermore, analysis of the top clones demonstrated a high degree of similarity within each group (**Figure 2D**). Notably, the occupied repertoire space corresponding to clonotype indices of 101–1000 was significantly lower in the MP group than in the HC group, whereas the repertoire space occupied by clonotype indices exceeding 1000 was significantly higher in the MP group (**Figure 2E**). These findings suggest distinct BCR repertoire characteristics in MP patients, with increased diversity and alterations in clonotype distribution.

### Preferential V and J gene usage in pediatric MP patients

**Figure 3:**
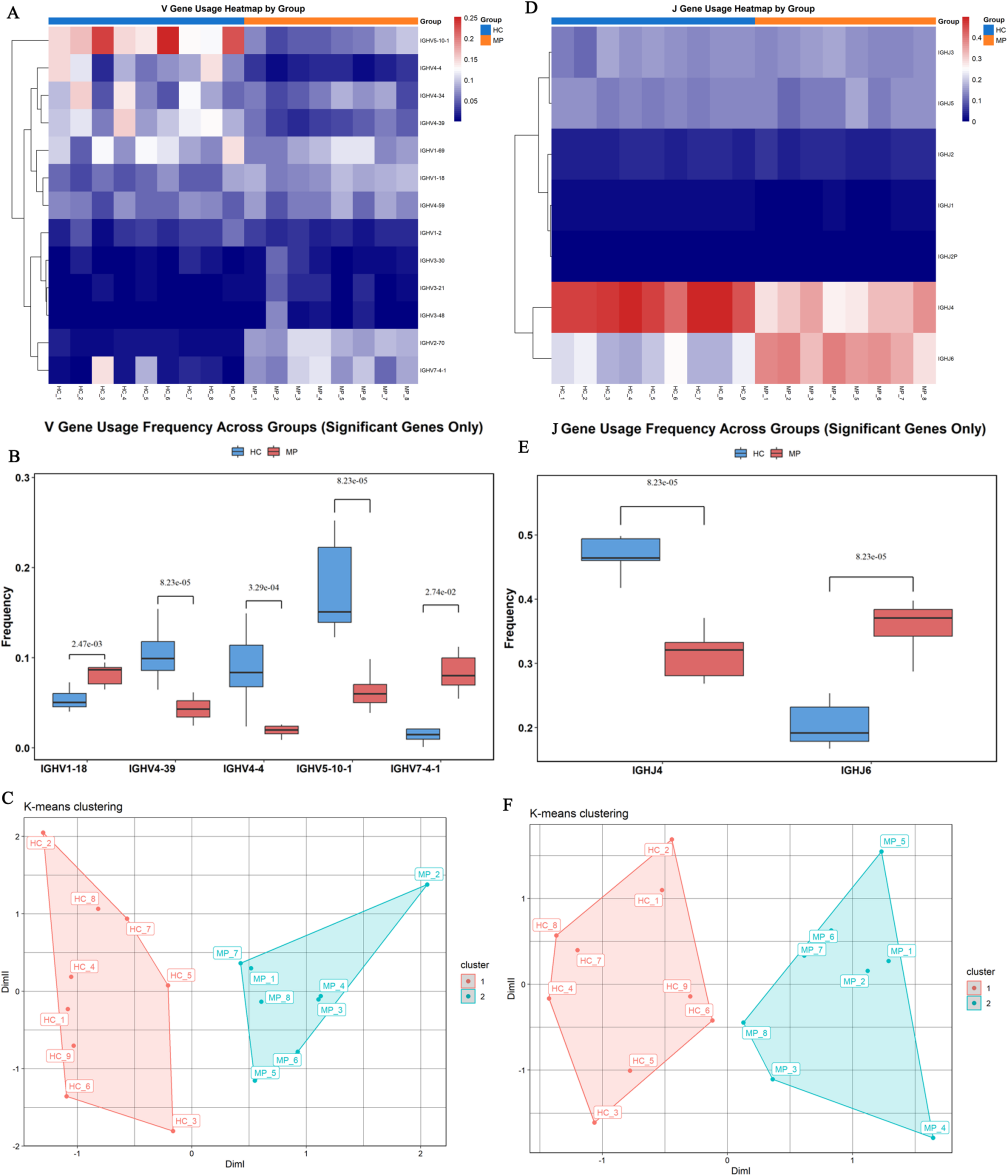
Preferential V and J gene usage. **(A)** Heatmap visualization of the top 13 most frequently used V genes across 17 samples. **(B)** Significant differences in V gene usage of top 13 most frequently used V genes. **(C)** Principal component analysis based on V gene usage showed clear separation between MP and HC samples. **(D)** Heatmap of J gene usage frequencies across all samples. **(E)** Identified significant differences in J gene usage. **(F)** Principal component analysis based on J gene usage demonstrated a clear distinction between MP and HC groups. MP, Mycoplasma pneumoniae; HC, Healthy control. Data are presented as mean ± SEM. Statistical significance was determined using unpaired two-tailed Student’s t-test. P < 0.05 indicates statistical difference.

To determine whether specific V or J gene segments exhibit preferential usage in pediatric MP patients, we analyzed their involvement in clonotype patterns compared to healthy controls. A total of 96 V genes were detected, and after filtering out those with low detection rates, we visualized the top 13 most frequently used V genes across all 17 samples using a heatmap (**Figure 3A**). The heatmap demonstrated distinct selective usage patterns of V genes between MP and HC groups, suggesting preferential selection of specific V segments in MP patients.

Further comparative analysis identified significant differences in the usage frequencies of IGHV1-18, IGHV4-39, IGHV4-4, IGHV5-10-1, and IGHV7-4–1 between the two groups (**Figure 3B**). Specifically, IGHV1–18 and IGHV7-4–1 were significantly enriched in the MP group compared to the HC group, whereas IGHV4-39, IGHV4-4, and IGHV5-10–1 showed significantly lower usage in MP patients. Principal component analysis (PCA) based on V gene usage frequencies further demonstrated a clear separation between MP and HC samples (**Figure 3C**).

Similarly, analysis of J gene usage revealed seven detectable J segments (**Figure 3D**). Among them, five J genes exhibited comparable usage frequencies between the two groups, whereas IGHJ4 was significantly underrepresented in the MP group, and IGHJ6 was significantly overrepresented in MP patients compared to HC (**Figure 3E**). PCA analysis based on J gene usage frequencies also showed a clear distinction between MP and HC groups, further reinforcing the presence of disease-associated differences in BCR repertoire composition (**Figure 3F**).

### CDR3 length distribution and amino acid usage in pediatric MP patients

**Figure 4:**
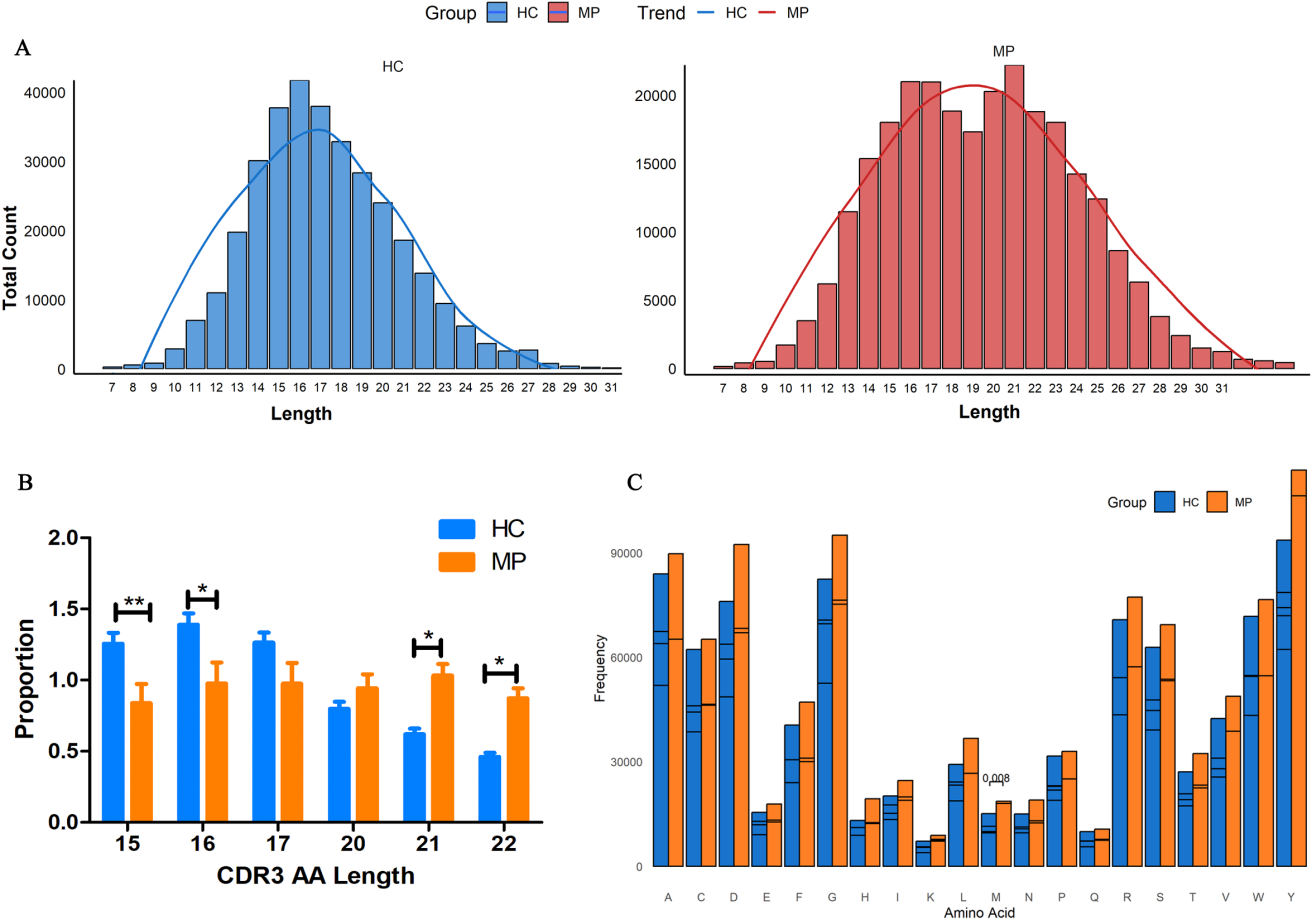
Altered CDR3 length distribution and amino acid usage. **(A)**CDR3 length distribution analysis revealed a conventional bell-shaped pattern in HC, whereas MP patients exhibited a bimodal distribution with an additional peak at 20–22 amino acids. **(B)** Quantification of CDR3 clonotypes at key length intervals. **(C)** Amino acid usage analysis within CDR3 regions between MP and HC groups. MP, Mycoplasma pneumoniae; HC, Healthy control. Data are presented as mean ± SEM. Statistical significance was determined using unpaired two-tailed Student’s t-test. P < 0.05 indicates statistical difference. *p < 0.05, **p < 0.001.

To further investigate the structural characteristics of the BCR heavy chain CDR3 repertoire, we analyzed the CDR3 length distribution and amino acid usage in MP patients and HC. The length distribution of unique CDR3 clonotypes exhibited distinct patterns between the two groups (**Figure 4A**). As expected, the HC group displayed a conventional bell-shaped distribution, with a peak at 15–17 amino acids. Interestingly, the MP group exhibited a bimodal distribution, with one peak at 15–17 amino acids, similar to the HC group, and an additional peak at 20–22 amino acids. Moreover, the average CDR3 length was significantly longer in MP patients (19.63 amino acids) compared to HC (17.27 amino acids), suggesting an altered selection process in the MP immune response.

Further quantitative analysis of CDR3 clonotype proportions at key length intervals revealed significant differences between the groups (**Figure 4B**). The proportion of CDR3 clonotypes with 15 and 16 amino acids was significantly higher in the HC group than in MP patients. In contrast, MP patients exhibited a significantly higher proportion of 21 and 22 amino acid-long CDR3 clonotypes, reinforcing the presence of an extended CDR3 repertoire in MP. Additionally, we analyzed amino acid usage within the CDR3 regions of both groups (**Figure 4C**). While the overall amino acid composition was largely similar, the frequency of methionine (M) usage was significantly different between MP and HC groups. These findings suggest that MP patients exhibit a distinct BCR repertoire characterized by an expanded CDR3 length distribution and altered amino acid usage, which may contribute to disease-associated immune responses.

### Disease-specific clonotypes in MP patients

**Figure 5:**
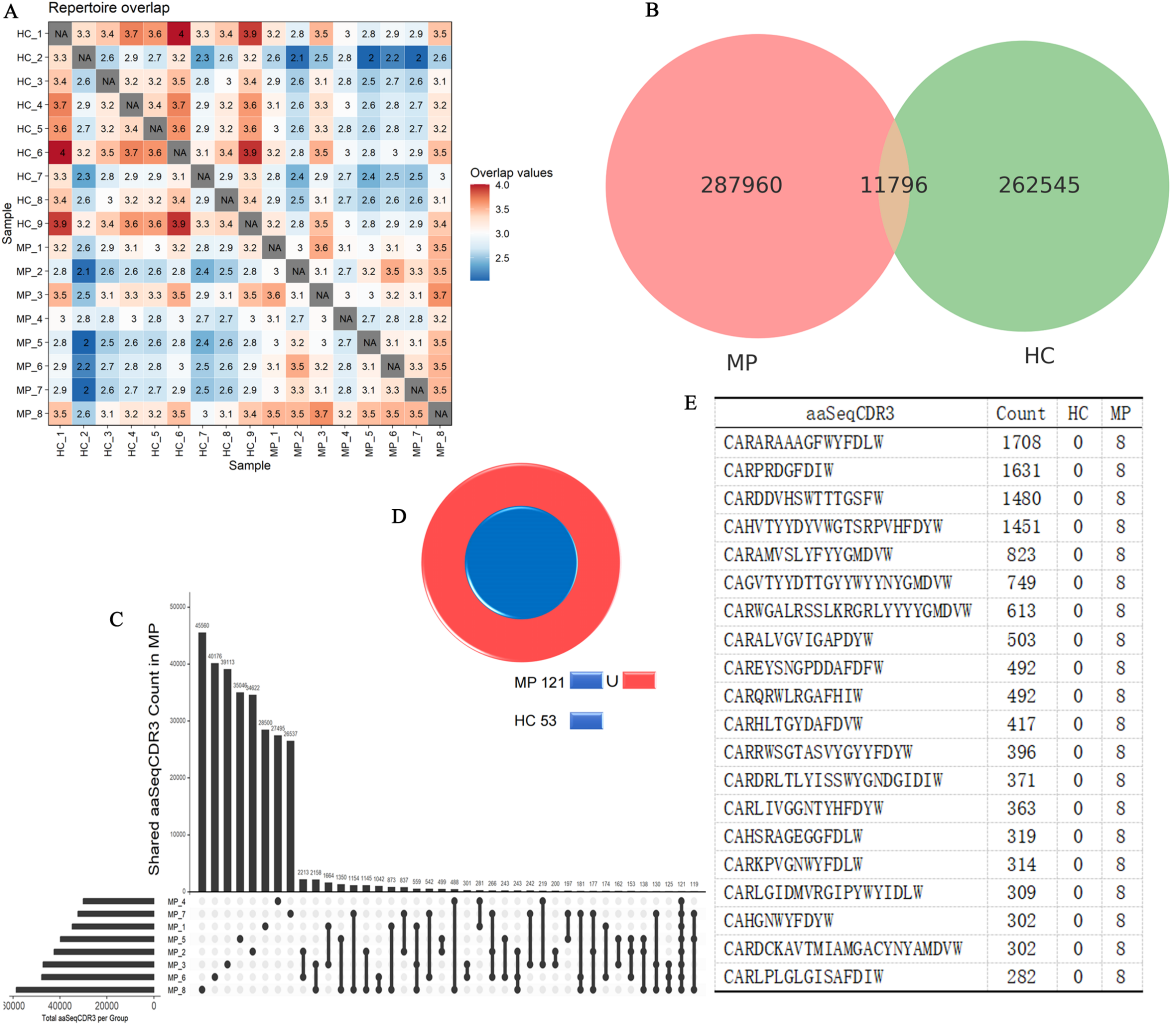
Disease-specific BCR clonotypes in MP patients. **(A)** Heatmap of BCR repertoire overlap among MP and HC individuals. **(B)** UpSet plot illustrating shared and unique clonotypes between MP and HC groups. **(C)** UpSet plot illustrating shared and unique clonotypes among MP cohort. **(D)** Cross-referencing analysis identified 68 clonotypes exclusive to MP patients. **(E)** The top 20 expanded clonotypes that are shared among MP patients and absent in the HC group. MP, Mycoplasma pneumoniae; HC, Healthy control.

To further explore the clonal architecture of the BCR repertoire in MP patients, we analyzed the degree of repertoire overlap among different samples (**Figure 5A**). The repertoire overlap heatmap revealed that certain clonotypes were shared among individuals, with the highest level of clonal sharing observed within the HC group, followed by the MP group. This suggests that healthy individuals maintain a more conserved BCR repertoire, whereas MP patients exhibit a more individualized immune response with distinct clonotypic expansions. To quantify the extent of shared and unique clonotypes between the two groups, we constructed an UpSet plot (**Figure 5B**), which demonstrated that 11,796 clonotypes were shared between MP and HC groups. This substantial overlap suggests the presence of a common baseline BCR repertoire that exists in both healthy and diseased states.

Next, we specifically examined intra-group clonal sharing within the MP cohort using another UpSet plot (**Figure 5C**). The results showed that the highest degree of sharing between two MP samples reached 2,213 clonotypes, while the maximum number of clonotypes shared across three MP samples was 559. Notably, a core set of 121 clonotypes was detected in all MP samples, suggesting a potential disease-associated repertoire.

To determine whether these 121 MP-specific clonotypes were also present in the HC group, we cross-referenced them against the HC dataset (**Figure 5D**). Among these, 53 clonotypes were also found in HC samples, leaving 68 clonotypes that were exclusively shared among MP patients but absent in HC individuals. These MP-specific clonotypes could represent disease-associated B cell expansions or antigen-driven immune responses unique to MP pathology. Further analysis of the 68 MP-exclusive clonotypes revealed substantial clonal amplification, suggesting their potential involvement in the immune response against MP. Among these, **Figure 5E** highlights the top 20 expanded clonotypes, with four clonotypes exhibiting extreme expansion (clone count >1,000). The most highly expanded clonotypes included: CARARAAAGFWYFDLW, CARPRDGFDIW, CARDDVHSWTTTGSFW, CAHVTYYDYVWGTSRPVHFDYW. To investigate whether these sequences shared common structural features, we performed motif analysis. Two conserved amino acid motifs, GNYGFDVW and CARARV, were identified among the dominant CDR3 sequences (**Supplementary Figure S1**). These motifs may reflect structural or functional convergence driven by antigen selection. The presence of such motifs, along with highly expanded clones, supports the notion of an antigen-driven B cell response in MP, potentially offering insights into MP-specific immunological memory or diagnostic biomarkers.

## Discussion

The composition and diversity of the BCR repertoire play a crucial role in shaping the humoral immune response and influencing disease outcomes following bacterial infections (Hoehn et al., 2016; Guo et al., 2019). Previous studies have highlighted dynamic alterations in the BCR repertoire in response to various pathogens, such as Streptococcus pneumoniae (Morgan et al., 2024) and Haemophilus influenzae (Truck et al., 2015). Moreover, the expansion and persistence of specific BCR clonotypes during chronic infections illustrate the evolving nature of the adaptive immune response (Florova et al., 2024; Jin et al., 2024). Our study demonstrates significant changes in the BCR repertoire of MP-infected children, suggesting that Mycoplasma pneumoniae infection induces a highly dynamic B cell response. Understanding BCR diversity and clonal expansion in this context may provide valuable insights into disease severity, immune protection, and potential therapeutic strategies.

Our findings demonstrate a significant increase in total B cell counts and serum immunoglobulin levels in MP-infected patients, indicating an enhanced humoral immune response. This is consistent with previous studies suggesting that MP infection triggers robust B cell activation and antibody production as a critical component of host defense (Simecka et al., 1993; Meyer Sauteur et al., 2020). Notably, we observed abnormalities in immunoglobulin levels, with IgM showing the most significant changes. This predominance of IgM elevation may be due to the early and primary nature of the humoral immune response against Mycoplasma pneumoniae infection. Since IgM is the first antibody isotype produced during an initial immune response (Jones et al., 2020), its higher levels likely reflect active B cell stimulation in response to persistent MP antigens (Dong et al., 2017). However, fewer abnormalities were observed in IgG and IgA levels, likely due to their limited antigen exposure history and immature germinal center reactions, which delay efficient IgG class-switching. In contrast, adults typically mount faster IgG-dominated responses owing to pre-existing memory B cells and well-developed secondary lymphoid structures (Semmes et al., 2020).

BCR repertoire analysis revealed significant differences in diversity indices between MP-infected and control groups. Notably, the BCR diversity in MP patients exhibited an overall increase, as reflected by the higher D50 index. This finding contrasts with the commonly observed decrease in diversity following SARS-CoV-2 (Xiao et al., 2024) or Pneumocystis (Sun et al., 2021) infections, where immune responses are often characterized by a contraction of the repertoire due to clonal expansion of pathogen-specific B cells. This could suggest that MP infection triggers the activation of a wider range of B cell clones, potentially to generate antibodies against a diverse array of epitopes on the pathogen. Further investigations into the specific characteristics of these expanded B cell clones, including their functional roles and antigen specificity, are needed to better understand the nature of the immune response in MP-infected individuals and how it differs from other bacterial or viral infections.

Our study identified distinct V and J gene usage patterns in MP-infected patients compared to healthy controls. Several V gene families, including IGHV1-18, IGHV3–23 and IGHV7-4, exhibited significantly higher usage frequencies, suggesting preferential selection in response to MP infection. The enrichment of these specific V gene families aligns with previous studies reporting their involvement in immune responses against pathogens. For example, IGHV1–18 has been identified as the germline origin of HmAb64, an antibody with a 15-amino-acid CDR H3, which neutralized 10% (20 out of 208) of cross-clade HIV-1 pseudoviruses, including tier 2 strains from clades B, BC, C, and G (Wang et al., 2024). Additionally, IGHV1–18 usage was significantly elevated in human B cells following dengue virus infection (Godoy-Lozano et al., 2016). IGHV3-23-IGHJ4 pairing has been associated with a monoclonal response specific to SARS-CoV-2 infection (Wen et al., 2020). Transcriptomic analysis of mixed peripheral blood mononuclear cells from moderate and severe SARS-CoV-2 patients revealed a significant upregulation of IGHV7–4 expression (Khalid et al., 2022). Furthermore, the observed differences in J gene segment usage, particularly the increased utilization of IGHJ6 and decreased utilization of IGHJ4. Both IGHJ4 and IGHJ6 have been reported to exhibit CDR-H3 loop length-dependent usage in the natural human immune repertoire (Wang et al., 2021), indicating their involvement in shaping antigen specificity. Notably, IGHJ6 has also been reported to be highly utilized in patients with SARS-CoV-2 infection (Zhou et al., 2021). Interestingly, while previous studies have shown that IGHJ4 is preferentially utilized across various immune-related conditions, including autoimmune diseases (Hirokawa et al., 2019), cancers (Katsibardi et al., 2011), and infectious diseases (Andreano et al., 2021), our study found a distinct pattern. Although IGHJ4 still exhibited biased usage in MP-infected patients, its overall frequency was lower than in healthy controls. Further investigations are needed to determine whether the altered IGHJ4 and IGHJ6 usage impacts BCR functionality and contributes to the immune response differences observed in MP infection.

The value of CDR3 length analysis lies in its ability to reflect the diversity of the TCR and Ig repertoire, revealing immune clone distribution patterns across individuals and over time (Miqueu et al., 2007). In this study, CDR3 length distribution analysis revealed a shift towards longer CDR3 sequences in MP-infected patients, which is commonly associated with increased antigen specificity and affinity maturation. For example, in IgG4-related disease (IgG4-RD), the length distribution of IgG4-RD-specific CDR3 amino acid sequences was skewed towards longer fragments (Wang et al., 2019). Notably, ultra-long CDR3 regions have been observed in certain species, such as cattle, where extensive somatic hypermutation generates a high degree of diversity. These long CDR3 sequences enable cattle to mount particularly strong immune responses against viral antigens, including broadly neutralizing responses to stable HIV gp140 trimers (Stanfield et al., 2018; Deiss et al., 2019). The shift towards longer CDR3 sequences in MP-infected patients observed in our study may similarly reflect an adaptive mechanism to enhance antigen recognition and immune response effectiveness.

During pathogen infection, the expansion and proliferation of BCRs are driven by interactions with pathogen-specific antigens (Tian et al., 2017; St John and Rathore, 2019). The immune response is triggered by both bacterial proteins and host-derived factors, leading to the selective proliferation of specific BCR clonotypes (Ngono and Shresta, 2018; Lee et al., 2022). The key finding of our study is the identification of disease-specific clonotypes that were significantly enriched in MP-infected patients. Several high-frequency clonotypes were exclusively detected in the MP group, suggesting their potential role as biomarkers for MP infection. The persistence of these clonotypes in multiple patients implies a conserved immune response to MP, which may be leveraged for diagnostic or therapeutic purposes. Additionally, the expanded clones exhibited convergent recombination patterns, indicating a shared immune response across individuals (Nielsen et al., 2020). These results highlight the potential of BCR repertoire analysis for identifying infection-specific immune signatures and advancing our understanding of host-pathogen interactions in MP infection.

Despite providing valuable insights into the IGH CDR3 repertoire of MP-infected patients, several limitations must be acknowledged to contextualize our findings. First, the sample size in this study was relatively small, which may limit the statistical power and generalizability of the results. Additionally, our study was cross-sectional, which restricts the ability to assess the temporal dynamics of TCR and Ig repertoires over the course of infection. Longitudinal studies are needed to track changes in immune repertoire diversity and its correlation with clinical outcomes over time. Addressing these limitations in future research would improve the robustness of the findings and provide a more comprehensive understanding of immune responses in MP infection.

In conclusion, our study provides novel insights into the humoral immune response to *Mycoplasma pneumoniae* infection by integrating serological markers, B cell counts, and BCR heavy chain CDR3 repertoire analysis. We identified increased B cell activation, elevated inflammatory and immunoglobulin markers, and significant changes in BCR diversity, gene usage preferences, CDR3 length distribution, and disease-specific clonotypes. These alterations, particularly the identification of MP-exclusive clonotypes and biased usage of IGHV and IGHJ genes, enhance our understanding of the immunopathology of MP infection. Importantly, these findings may contribute to the development of improved diagnostic and therapeutic strategies. For instance, the MP-specific public clonotypes or stereotyped BCR features identified in this study may serve as potential biomarkers for early diagnosis or disease monitoring. Additionally, repertoire characteristics associated with disease could inform precision immunotherapy design and provide novel targets for B cell–modulating interventions in pediatric MP.

## Data Availability

The datasets presented in this study can be found in online repositories. The names of the repository/repositories and accession number(s) can be found in the article/**Supplementary Material**.
