# Supplementary figures and images for: Alterations in BCR heavy chain CDR3 repertoire characteristics in pediatric mycoplasma pneumoniae infection
Source: Front Cell Infect Microbiol. 2025 Jun 12;15:1573511. doi: 10.3389/fcimb.2025.1573511 (PMC12198128; doi:10.3389/fcimb.2025.1573511)

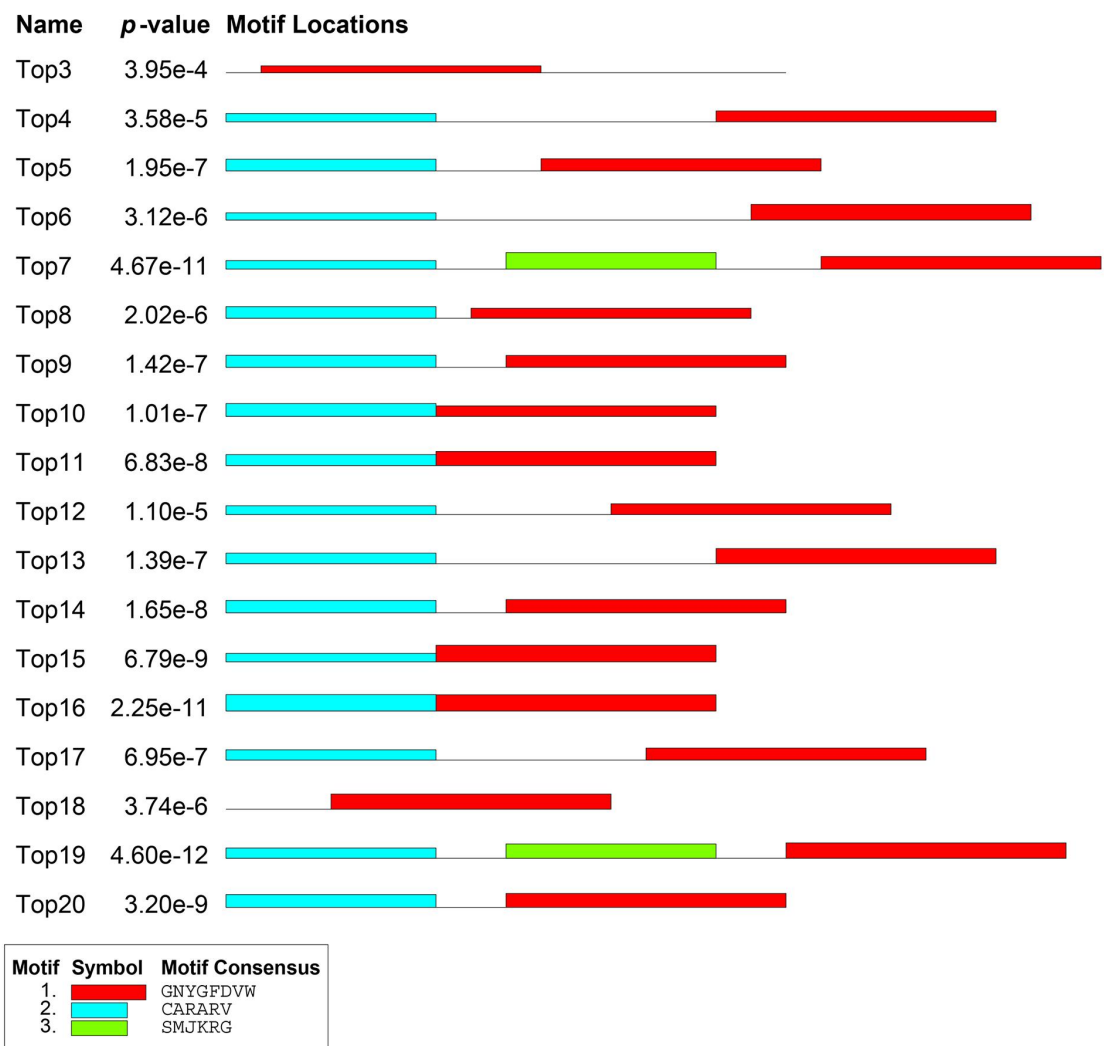

Figure S1. Motif discovery among the top 20 MP-specific CDRH3 sequences using MEME analysis.

Supplement: Supplementary file 1 [file DataSheet1.pdf]
